# 2-(Chloro­meth­yl)benzimidazolium chloride

**DOI:** 10.1107/S1600536809015359

**Published:** 2009-04-30

**Authors:** Gang Wang, Zhi-Rong Qu

**Affiliations:** a Ordered Matter Science Research Center, College of Chemistry and Chemical Engineering, Southeast University, Nanjing 210096, People’s Republic of China

## Abstract

The structure of title compound, C_8_H_8_ClN_2_^+^·Cl^−^, comprises discrete ions which are inter­connected by N—H⋯Cl hydrogen bonds, leading to a neutral one-dimensional network in [001]. This hydrogen bonding appears to complement π–π stacking inter­actions [centroid–centroid distances 3.768 (2) and 3.551 (2) Å] and helps to stabilize the structure further.

## Related literature

For details of the preparation of imidazole compounds, see: Ikezaki & Nakamura (2002 ▶). For the chemistry of 2-(chloro­meth­yl)-1*H*-benzo[*d*]imidazolium chloride, see: Jian *et al.* (2003 ▶).
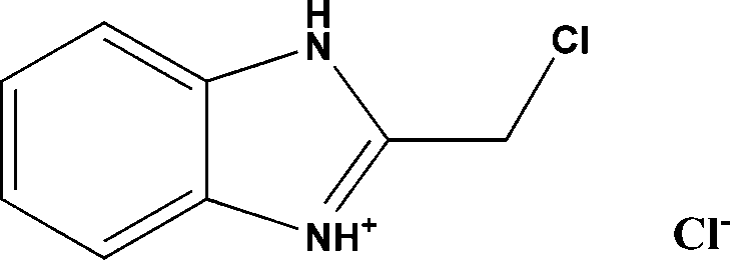


## Experimental

### 

#### Crystal data

C_8_H_8_ClN_2_^+^·Cl^−^
*M* *_r_* = 203.06
Monoclinic, 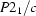

*a* = 7.1972 (14) Å
*b* = 9.4507 (19) Å
*c* = 14.046 (3) Å
β = 102.51 (3)°
*V* = 932.7 (3) Å^3^
*Z* = 4
Mo *K*α radiation
μ = 0.64 mm^−1^
*T* = 293 K
0.22 × 0.20 × 0.20 mm

#### Data collection

Rigaku SCXmini diffractometer
Absorption correction: multi-scan (*CrystalClear*; Rigaku, 2005 ▶) *T*_min_ = 0.867, *T*_max_ = 0.882
9462 measured reflections
2141 independent reflections
1212 reflections with *I* > 2σ(*I*)
*R*_int_ = 0.083
Standard reflections: ?

#### Refinement

*R*[*F* ^2^ > 2σ(*F* ^2^)] = 0.056
*wR*(*F* ^2^) = 0.180
*S* = 0.84
2141 reflections
109 parameters
.
Δρ_max_ = 0.29 e Å^−3^
Δρ_min_ = −0.31 e Å^−3^

### 

Data collection: *CrystalClear* (Rigaku, 2005 ▶); cell refinement: *CrystalClear*; data reduction: *CrystalClear*; program(s) used to solve structure: *SHELXS97* (Sheldrick, 2008 ▶); program(s) used to refine structure: *SHELXL97* (Sheldrick, 2008 ▶); molecular graphics: *SHELXTL/PC* (Sheldrick, 2008 ▶); software used to prepare material for publication: *PRPKAPPA* (Ferguson, 1999 ▶).

## Supplementary Material

Crystal structure: contains datablocks I, global. DOI: 10.1107/S1600536809015359/bx2205sup1.cif

Structure factors: contains datablocks I. DOI: 10.1107/S1600536809015359/bx2205Isup2.hkl

Additional supplementary materials:  crystallographic information; 3D view; checkCIF report

## Figures and Tables

**Table 1:** Hydrogen-bond geometry (Å, °)

*D*—H⋯*A*	*D*—H	H⋯*A*	*D*⋯*A*	*D*—H⋯*A*
N1—H1*A*⋯Cl1^i^	0.86	2.25	3.066 (2)	158
N2—H2*A*⋯Cl1	0.86	2.20	3.055 (2)	178

Symmetry code: (i) 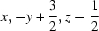.

**Fig. 1.**
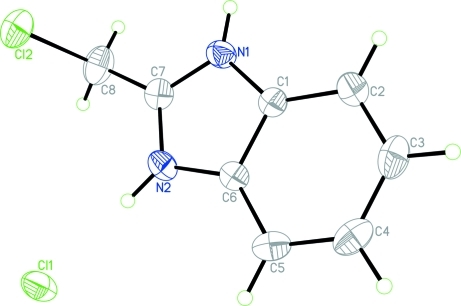
The molecular structure of (I), with the displacement ellipsoids were drawn at the 30% probability level.

## supplementary crystallographic information

### Comment

2-(4-bromophenyl)-1-phenyl-1*H*-benzimidazole used as bridging ligands in coordination and metallosupramolecular chemistry are representative. In recent years, benzimidazole also was used to link different alkyl or aromatic group, which can adopt different conformations according to the different geometric requirements of metal centers when forming metal complexes (Ikezaki, *et al.* 2002; Jian, *et al.* 2003). We report here the crystal structure of the title compound. The structure of title compound, C_8_H_8_ClN_2_.Cl^-^, comprises discrete ions which are interconnected by N1—H1A···Cl1^i^ hydrogen bond, leading to a neutral one-dimensional network in [0 0 1] direction. These hydrogen bonds appear to complement π-π stacking interactions and help to stabilize the structure further (Table 2).

### Experimental

A mixture of 1,2-diaminobenzene (0.01 mol 1.08 g) and chloroacetic acid (0.01 mol 0.95 g) in HCl (4 ml) was refluxed for 12 h and the title compound was dissolved in ethanol and HCl, after slowly volatilizing over a period of 48 h, colorless crystals of the title compound suitable for diffraction were isolated.

### Refinement

Positional parameters of all the H atoms were calculated geometrically and were allowed to ride on the C, N atoms to which they are bonded, with C—H = 0.93 to 0.97 Å, *U*_iso_(H) = 1.2 *U*_eq_(C), N—H = 0.86 Å, *U*_iso_(H) = 1.2 *U*_eq_(N).

### Crystal data

**Table:**

C_8_H_8_ClN_2_^+^·Cl^−^	*F*(000) = 416
*M**_r_* = 203.06	*D*_x_ = 1.446 Mg m^−^^3^
Monoclinic, *P*2_1_/*c*	Mo *K*α radiation, λ = 0.71073 Å
Hall symbol: -p 2ybc	Cell parameters from 1979 reflections
*a* = 7.1972 (14) Å	θ = 3.1–27.5°
*b* = 9.4507 (19) Å	µ = 0.64 mm^−^^1^
*c* = 14.046 (3) Å	*T* = 293 K
β = 102.51 (3)°	Prism, colourless
*V* = 932.7 (3) Å^3^	0.22 × 0.20 × 0.20 mm
*Z* = 4	

### Data collection

**Table:**

Rigaku SCXmini diffractometer	2141 independent reflections
Radiation source: fine-focus sealed tube	1212 reflections with *I* > 2σ(*I*)
graphite	*R*_int_ = 0.083
CCD_Profile_fitting scans	θ_max_ = 27.5°, θ_min_ = 3.6°
Absorption correction: multi-scan (*CrystalClear*; Rigaku, 2005)	*h* = −9→9
*T*_min_ = 0.867, *T*_max_ = 0.882	*k* = −12→12
9462 measured reflections	*l* = −18→18

### Refinement

**Table:**

Refinement on *F*^2^	Primary atom site location: structure-invariant direct methods
Least-squares matrix: full	Secondary atom site location: difference Fourier map
*R*[*F*^2^ > 2σ(*F*^2^)] = 0.056	Hydrogen site location: inferred from neighbouring sites
*wR*(*F*^2^) = 0.180	*w* = 1/[σ^2^(*F*_o_^2^) + (0.1077*P*)^2^ + 0.4199*P*] where *P* = (*F*_o_^2^ + 2*F*_c_^2^)/3
*S* = 0.84	(Δ/σ)_max_ < 0.001
2141 reflections	Δρ_max_ = 0.29 e Å^−^^3^
109 parameters	Δρ_min_ = −0.31 e Å^−^^3^
0 restraints	

### Special details

**Table:**

Geometry. All e.s.d.'s (except the e.s.d. in the dihedral angle between two l.s. planes) are estimated using the full covariance matrix. The cell e.s.d.'s are taken into account individually in the estimation of e.s.d.'s in distances, angles and torsion angles; correlations between e.s.d.'s in cell parameters are only used when they are defined by crystal symmetry. An approximate (isotropic) treatment of cell e.s.d.'s is used for estimating e.s.d.'s involving l.s. planes.
Refinement. Refinement of *F*^2^ against ALL reflections. The weighted *R*-factor *wR* and goodness of fit *S* are based on *F*^2^, conventional *R*-factors *R* are based on *F*, with *F* set to zero for negative *F*^2^. The threshold expression of *F*^2^ > σ(*F*^2^) is used only for calculating *R*-factors(gt) *etc*. and is not relevant to the choice of reflections for refinement. *R*-factors based on *F*^2^ are statistically about twice as large as those based on *F*, and *R*- factors based on ALL data will be even larger.

### Fractional atomic coordinates and isotropic or equivalent isotropic displacement parameters (Å^2^)

**Table:**

	*x*	*y*	*z*	*U*_iso_*/*U*_eq_	
Cl1	0.34571 (17)	0.59201 (10)	1.15934 (7)	0.0608 (4)	
Cl2	0.11234 (16)	0.56643 (10)	0.83757 (8)	0.0654 (4)	
N2	0.2890 (4)	0.8248 (3)	1.00495 (19)	0.0449 (7)	
H2A	0.3032	0.7611	1.0496	0.054*	
N1	0.2626 (4)	0.9234 (3)	0.86427 (19)	0.0446 (7)	
H1A	0.2575	0.9338	0.8029	0.053*	
C6	0.2574 (4)	0.9674 (4)	1.0180 (2)	0.0385 (8)	
C1	0.2393 (5)	1.0306 (3)	0.9280 (2)	0.0388 (8)	
C7	0.2936 (5)	0.8027 (4)	0.9125 (3)	0.0438 (8)	
C5	0.2467 (5)	1.0453 (4)	1.1012 (3)	0.0528 (10)	
H5A	0.2596	1.0028	1.1620	0.063*	
C2	0.2079 (5)	1.1754 (4)	0.9153 (3)	0.0528 (10)	
H2B	0.1957	1.2183	0.8546	0.063*	
C4	0.2163 (6)	1.1877 (4)	1.0883 (3)	0.0588 (11)	
H4A	0.2090	1.2435	1.1419	0.071*	
C3	0.1961 (6)	1.2515 (4)	0.9973 (3)	0.0615 (11)	
H3A	0.1738	1.3484	0.9917	0.074*	
C8	0.3276 (6)	0.6648 (4)	0.8695 (3)	0.0628 (11)	
H8A	0.4205	0.6114	0.9162	0.075*	
H8B	0.3787	0.6801	0.8119	0.075*	

### Atomic displacement parameters (Å^2^)

**Table:**

	*U*^11^	*U*^22^	*U*^33^	*U*^12^	*U*^13^	*U*^23^
Cl1	0.0894 (8)	0.0515 (6)	0.0475 (6)	0.0166 (5)	0.0280 (5)	0.0084 (4)
Cl2	0.0670 (7)	0.0524 (6)	0.0753 (7)	−0.0038 (5)	0.0119 (5)	−0.0149 (5)
N2	0.0488 (18)	0.0411 (16)	0.0452 (17)	0.0062 (14)	0.0111 (14)	0.0093 (12)
N1	0.0527 (19)	0.0459 (17)	0.0377 (15)	0.0017 (14)	0.0154 (14)	0.0019 (13)
C6	0.0319 (18)	0.0439 (18)	0.0407 (18)	0.0021 (15)	0.0104 (14)	0.0029 (14)
C1	0.0361 (18)	0.0403 (17)	0.0409 (18)	−0.0009 (15)	0.0105 (15)	−0.0002 (15)
C7	0.0383 (19)	0.0436 (19)	0.052 (2)	0.0025 (16)	0.0157 (16)	−0.0010 (16)
C5	0.050 (2)	0.071 (3)	0.0383 (19)	0.002 (2)	0.0099 (16)	−0.0059 (17)
C2	0.058 (2)	0.046 (2)	0.054 (2)	0.0001 (18)	0.0104 (19)	0.0072 (17)
C4	0.054 (2)	0.064 (3)	0.057 (2)	0.005 (2)	0.011 (2)	−0.022 (2)
C3	0.061 (3)	0.042 (2)	0.082 (3)	0.005 (2)	0.015 (2)	−0.013 (2)
C8	0.053 (2)	0.051 (2)	0.087 (3)	0.002 (2)	0.021 (2)	−0.016 (2)

### Geometric parameters (Å, °)

**Table:**

C1—C6	1.381 (4)	C5—H5A	0.9300
C1—C2	1.389 (4)	C6—N2	1.385 (4)
C1—N1	1.391 (3)	C7—N2	1.320 (3)
C2—C3	1.372 (4)	C7—N1	1.322 (3)
C2—H2B	0.9300	C7—C8	1.477 (4)
C3—C4	1.395 (4)	C8—Cl2	1.781 (3)
C3—H3A	0.9300	C8—H8A	0.9700
C4—C5	1.367 (4)	C8—H8B	0.9700
C4—H4A	0.9300	N1—H1A	0.8600
C5—C6	1.392 (4)	N2—H2A	0.8600
			
C6—C1—C2	121.8 (3)	N2—C6—C5	132.0 (3)
C6—C1—N1	106.0 (2)	N2—C7—N1	109.3 (2)
C2—C1—N1	132.2 (3)	N2—C7—C8	125.5 (3)
C3—C2—C1	116.4 (3)	N1—C7—C8	125.2 (3)
C3—C2—H2B	121.8	C7—C8—Cl2	110.6 (2)
C1—C2—H2B	121.8	C7—C8—H8A	109.5
C2—C3—C4	121.8 (3)	Cl2—C8—H8A	109.5
C2—C3—H3A	119.1	C7—C8—H8B	109.5
C4—C3—H3A	119.1	Cl2—C8—H8B	109.5
C5—C4—C3	121.9 (3)	H8A—C8—H8B	108.1
C5—C4—H4A	119.1	C7—N1—C1	109.1 (2)
C3—C4—H4A	119.1	C7—N1—H1A	125.5
C4—C5—C6	116.5 (3)	C1—N1—H1A	125.5
C4—C5—H5A	121.7	C7—N2—C6	109.2 (2)
C6—C5—H5A	121.7	C7—N2—H2A	125.4
C1—C6—N2	106.4 (2)	C6—N2—H2A	125.4
C1—C6—C5	121.5 (3)		
			
C6—C1—C2—C3	0.1 (5)	N2—C7—C8—Cl2	84.1 (4)
N1—C1—C2—C3	−178.5 (3)	N1—C7—C8—Cl2	−95.6 (3)
C1—C2—C3—C4	0.6 (5)	N2—C7—N1—C1	1.1 (3)
C2—C3—C4—C5	−0.7 (5)	C8—C7—N1—C1	−179.1 (3)
C3—C4—C5—C6	0.1 (5)	C6—C1—N1—C7	−0.3 (3)
C2—C1—C6—N2	−179.4 (3)	C2—C1—N1—C7	178.4 (3)
N1—C1—C6—N2	−0.5 (3)	N1—C7—N2—C6	−1.5 (3)
C2—C1—C6—C5	−0.7 (4)	C8—C7—N2—C6	178.8 (3)
N1—C1—C6—C5	178.2 (3)	C1—C6—N2—C7	1.2 (3)
C4—C5—C6—C1	0.6 (4)	C5—C6—N2—C7	−177.3 (3)
C4—C5—C6—N2	178.9 (3)		

### Hydrogen-bond geometry (Å, °)

**Table:**

*D*—H···*A*	*D*—H	H···*A*	*D*···*A*	*D*—H···*A*
N1—H1A···Cl1^i^	0.86	2.25	3.066 (2)	158
N2—H2A···Cl1	0.86	2.20	3.055 (2)	178

Symmetry codes: (i) *x*, −*y*+3/2, *z*−1/2.

### Table 2 π-π interaction in (I). α is dihedral angle between the planes, DCC is the length of the CC vector (centroid to centroid), τ is the angle(s) subtended by the plane normal(s) to CC. Cg1 is the centroid of ring N1, C1, C6, N2, C7, Cg2 of ring C1 C2 C3 C4 C5 C6.

**Table:**

Group 1	Group 2	α /°	DCC /Å	τ /°
Cg1	Cg2^i^	1.43	3.768 (2)	21.88
Cg1	Cg2^ii^	1.43	3.551 (2)	12.47

Symmetry codes: (i) -x, 2-y, 2-z (ii) 1-x, 2-y, 2-z
